# Aspirin Inhibits Colon Cancer Cell and Tumor Growth and Downregulates Specificity Protein (Sp) Transcription Factors

**DOI:** 10.1371/journal.pone.0048208

**Published:** 2012-10-26

**Authors:** Satya Pathi, Indira Jutooru, Gayathri Chadalapaka, Vijayalekshmi Nair, Syng-Ook Lee, Stephen Safe

**Affiliations:** 1 Department of Veterinary Physiology & Pharmacology, Texas A&M University, College Station, Texas, United States of America; 2 Institute of Biosciences and Technology, Texas A&M Health Science Center, Houston, Texas, United States of America; Wayne State University School of Medicine, United States of America

## Abstract

Acetylsalicylic acid (aspirin) is highly effective for treating colon cancer patients postdiagnosis; however, the mechanisms of action of aspirin in colon cancer are not well defined. Aspirin and its major metabolite sodium salicylate induced apoptosis and decreased colon cancer cell growth and the sodium salt of aspirin also inhibited tumor growth in an athymic nude mouse xenograft model. Colon cancer cell growth inhibition was accompanied by downregulation of Sp1, Sp3 and Sp4 proteins and decreased expression of Sp-regulated gene products including bcl-2, survivin, VEGF, VEGFR1, cyclin D1, c-MET and p65 (NFκB). Moreover, we also showed by RNA interference that β-catenin, an important target of aspirin in some studies, is an Sp-regulated gene. Aspirin induced nuclear caspase-dependent cleavage of Sp1, Sp3 and Sp4 proteins and this response was related to sequestration of zinc ions since addition of zinc sulfate blocked aspirin-mediated apoptosis and repression of Sp proteins. The results demonstrate an important underlying mechanism of action of aspirin as an anticancer agent and, based on the rapid metabolism of aspirin to salicylate in humans and the high salicylate/aspirin ratios in serum, it is likely that the anticancer activity of aspirin is also due to the salicylate metabolite.

## Introduction

Acetylsalicylic acid or aspirin is a non-steroidal anti-inflammatory drug (NSAID) widely used for treatment of pain, fever and other inflammatory conditions [1] and the role of aspirin and other NSAIDs in cancer has been extensively investigated [2], [3]. Aspirin use is associated with decreased risk for colorectal, breast, esophageal, lung, stomach and ovarian cancer, and aspirin is both a chemopreventive and chemotherapeutic agent for breast and colon cancer [4]–[8]. A recent report on the chemopreventive effects of aspirin showed that the incidence of colon cancer in Scotland was significantly decreased in the general population at the lowest daily dose of aspirin (75 mg) and the decreased incidence was observed even after only 5 yr of aspirin use [7]. In another study on the chemotherapeutic effects of aspirin in colon cancer patients, a hazard ratio of 0.53 (for mortality) was observed in patients who did not use the drug prior to diagnosis and this value decreased to 0.39 for a subset of patients overexpressing cyclooxygenase-2 (COX-2) [5].

Several studies on colon cancer cells and colon tumor models have confirmed that aspirin inhibits growth and induces apoptosis in these systems; however, the specific effects of aspirin are somewhat variable in these reports. For example, aspirin downregulates bcl-2 expression [9], inhibits vascular endothelial growth factor, exhibits antiangiogenic activity [10], [11], and inhibits the WNT/β-catenin pathway [12]. Dunlop and coworkers have also demonstrated that aspirin-induced downregulation of IκBα in colon cancer cells results in enhanced nuclear accumulation of the NFκB complex (p65/p50) and this has been linked to a pro-apoptotic pathway in colon cancer cells [13]–[15].

Ethyl 2-[(2,3-bis(nitrooxy)propyl)disulfanyl]benzoate (GT-094) is a synthetic nitro-non-steroidal anti-inflammatory drug (NO-NSAID) and like aspirin, GT-094 also inhibits colon cancer cell and tumor growth [16], [17]. Mechanistic studies indicate that the anticancer activity of GT-094 is due, in part, to ROS-dependent downregulation of specificity protein (Sp) transcription factors Sp1, Sp3, Sp4 and Sp-regulated genes which include bcl-2, survivin, hepatocyte growth factor receptor (c-MET), VEGF and its receptor VEGFR1 [17]. Other drugs including NSAIDs such as tolfenamic acid and COX-2 inhibitors also inhibit cancer cell growth and downregulate Sp transcription factors [18]–[26] and, in this study, we have investigated the effects of aspirin on Sp proteins and other Sp-regulated genes including β-catenin. Our results show that aspirin and salicylate downregulate Sp1, Sp3, Sp4 and several Sp-regulated gene products in colon cancer cells and identifies an important pathway for the anticancer activity of aspirin that is consistent with RNA interference (RNAi) studies in which knockdown Sp1, Sp3 and Sp4 in cancer cells also inhibits growth and induces apoptosis [24]–[26]. Knockdown of Sp proteins also demonstrated that β-catenin is an Sp-regulated gene in colon cancer cells. Results of this study, coupled with previous reports on the mechanisms of aspirin-mediated inhibition of colon cancer cell growth, will also facilitate development of therapies with aspirin and NSAID analogs in combination with other agents used to treat colon cancer. The reported high serum salicylate/aspirin ratios observed in human studies using aspirin [27] suggest that salicylate may be an important contributor to the anticancer activity of aspirin in colon cancer patients.

## Experimental Procedures

### Cell lines, reagents and antibodies

RKO, SW480, HT-29 and HCT-116 human colon carcinoma cell lines were obtained from American Type Culture Collection (Manassas, VA). RKO and SW480 cells were maintained in Dulbecco's modified/Ham's F-12 (Sigma-Aldrich, St. Louis, MO) with phenol red supplemented with 0.22% sodium bicarbonate, 5% fetal bovine serum, and 10 ml/L 100X antibiotic/antimycotic solution (Sigma). HT-29 and HCT-116 cells were maintained in McCoy's 5A medium (Sigma-Aldrich, St. Louis, MO) with phenol red supplemented with 0.22% sodium bicarbonate, 10% fetal bovine serum, and 10 ml/L 100X anti-biotic anti-mycotic solution (Sigma). The cells were grown in 150 cm^2^ culture plates in an air/CO_2_ (95∶5) atmosphere at 37°C and passaged approximately every 3–5 days. All antibodies were purchased from Santa Cruz Biotechnology (Santa Cruz, CA), except cleaved poly (ADP) ribose polymerase (PARP) and c-Met (Cell Signaling Technology, Danvers, MA), Sp1, survivin and VEGF-R2 (Millipore, Temecula, CA), VEGFR1 and p65 (Abcam Inc. Cambridge, MA), and β-actin antibodies (Sigma-Aldrich). β-Catenin was purchased from Epitomics, Inc., Burlingame, CA. The NSAIDs acetylsalicylic acid and sodium salicylate were purchased from Sigma-Aldrich. N,N,N',N'-Tetrakis(2-pyridyl methyl)ethylenediamine (TPEN), glutathione (98%) (GSH), and lactacystin were purchased from Sigma-Aldrich (St Louis, MO). Dithiothretol (DTT) (98%) was obtained from Boehringer Mannheim Corp, (Indianapolis, IN). Caspase inhibitors 2, 8 and 9 and pancaspase inhibitor (Z-VAD-fmk) are purchased from Calbiochem (San Diego, CA). Leptomycin B was inhibitor of nuclear export purchased from Enzo Life Sciences International, Inc. (Plymouth Meeting, PA). Lipofectamine and Lipofectamine 2000 were purchased from Invitrogen. Luciferase reagent was from Promega (Madison, WI). β-Galactosidase reagent was obtained from Tropix (Bedford, MA). NFκB promoter construct was purchased from Stratagene (Cedar Creek, TX). The VEGF and survivin promoter constructs were provided by Drs. Gerhard Siemeister and Gunter Finkenzeller (Institute of Molecular Medicine, Tumor Biology Center, Freiburg, Germany) and Dr. M. Zhou (Emory university, Atlanta, GA), respectively. Sp1 and Sp3 promoter constructs were kindly provided by Drs. Carlos Cuidad and Veronique Noe (University of Barcelona, Barcelona, Spain).

### Cell proliferation assays

RKO, SW480, HT-29 and HCT-116 colon cancer cell lines were plated (3×10^4^ per well) using DMEM:Ham's F-12 medium containing 2.5% charcoal stripped fetal bovine serum (FBS) in 12-well plates and left to attach for 24 hr. Cells were then treated with either vehicle or the indicated concentrations of aspirin and sodium salicylate. After 24, 48 and 72 hr of treatment, cells were counted using a Coulter Z1 particle counter. Each experiment was carried out in triplicate and results are expressed as means ± SE for each determination.

### Annexin V staining

Apoptosis, necrotic and healthy cell detection kit was purchased from Biotium, Inc (Hayward, CA). RKO, SW480, HT-29 and HCT-116 colon cancer cells (7.5×10^4^) were seeded in Lab-Tek two chambered cover glass slides and allowed to attach overnight. After treatment with aspirin (10 mM) for 24 hr, cells were washed with cold phosphate-buffered saline (PBS) twice and incubated with FITC Annexin V, ethidium homodimer III and Hoechst 33342 in Annexin V binding buffer for 20 min according to the manufacturer's instructions in the protocol. The cells were then washed twice with Annexin V binding buffer and detected for flouroscence with digital fluorescent microscope.

### siRNA interference assays

SiRNAs for Sp1, Sp3, Sp4, and Lamin were purchased from Sigma-Aldrich. The siRNA complexes used in this study are indicated as follows:

Lamin: SASI_Hs02_00367643

Sp1: SASI_Hs02_00363664

Sp3 5′-GCG GCA GGU GGA GCC UUC ACU TT

Sp4 5′-GCA GUG ACA CAU UAG UGA GCT T

RKO and SW480 colon cancer cell lines were seeded (6×10^4^ per ml) in 6-well plates in DMEM:Ham's F-12 medium supplemented with 2.5% charcoal-stripped FBS without antibiotic and left to attach for 1 d. Knockdown of Sp1, Sp3, Sp4 individually or a combination of all 3 proteins carried out using appropriate siRNA along with iLamin as control was performed using LipofectAMINE 2000 transfection reagent as per the manufacturer's instructions.

### Transfection and luciferase assay

RKO and SW480 colon cancer cells (1×10^5^ per well) were plated in 12-well plates in DMEM/Ham's F-12 medium supplemented with 2.5% charcoal-stripped FBS. After 24 hr, various amounts of DNA [i.e., 0.4 µg PGL3-Luc, 0.04 µg β-galactosidase, and 0.4 µg pNFκB-Luc (4)-Luc] were transfected using LipofectAMINE 2000 reagent according to the manufacturer's protocol. After 5 hr of the transfection, the transfection mix was replaced with complete medium containing either vehicle (DMSO) or the indicated compound in DMSO.

For RNA interference studies, RKO and SW480 colon cancer cells were cotransfected with siRNA for Sp1, Sp3, Sp4 or Lamin along with various amounts of DNA for PGL3-Luc or 0.4 µg pNFκB-Luc. After 22 hr, cells were then lysed with 100 µL of 1X reporter lysis buffer, and cell extracts (30 mL) were used for luciferase and β-galactosidase assays. A Lumicount luminometer was used to quantitate luciferase and β-galactosidase activities, and the luciferase activities were normalized to β-galactosidase activity.

### Western blots

RKO, SW480, HT-29 and HCT-116 colon cancer cells were seeded in DMEM:Ham's F-12 medium containing 2.5% charcoal-stripped FBS and, after 24 hr, cells were treated with either vehicle (DMSO) or the indicated compounds. Cells were collected using high-salt buffer (50 mM HEPES, 0.5 mol/L NaCl, 1.5 mM MgCl_2_, 1 mM EGTA, 10% glycerol, and 1% Triton-X-100) and 10 ml/L Protease Inhibitor Cocktail (Sigma-Aldrich). After centrifugation of the lysates at 15,000 *g* for 15 min at 4°C, the supernatants were recovered, and protein was quantified by the Bradford protein assay. Protein lysates (15–60 µg) were incubated for 5–10 min at 100°C along with 5X loading buffer and then separated by electrophoresis on 7.5–12% sodium dodecyl sulphate-polyacrylamide gels at 120 V for 3 to 4 hr. Proteins were transferred onto polyvinylidene difluoride membranes by wet electroblotting in a buffer containing 25 mM Tris, 192 mM glycine, and 20% methanol for 1.5 hr at 180 mÅ. Membranes were blocked for 45 min with 5% TBST-Blotto (10 mM Tris-HCl, 150 mM NaCl, pH 8.0, 0.05% Triton X-100, and 5% nonfat dry milk) and incubated in fresh 5% TBST-Blotto with 1∶500 primary antibody overnight with gentle shaking at 4°C. After washing twice with TBST for 10 min, the membrane was incubated with secondary antibody (1∶5000) in 5% TBST-Blotto for 3–4 hr by gentle shaking. The membrane was washed twice with TBST for 10 min, incubated with 2 ml of chemiluminescence substrate (Millipore, Temecula, CA) for 1 min, and exposed to Kodak image station 4000 mm Pro (Carestream Health, Rochester, NY).

### Xenograft studies in athymic mice

Female athymic nude mice were purchased from Harlan Laboratories (Indianapolis, IN). The mice were housed and maintained under specific pathogen-free conditions in facilities approved by the American Association for Accreditation of Laboratory Animal Care and in accordance with current regulations and standards of the United States Department of Agriculture, United States Department of Health and Human Services. This study was carried out in strict accordance with the recommendations in the Guide for the Care and Use of Laboratory Animals of the National Institutes of Health. The protocol was approved by the Texas A&M University Institutional Animal Care and Use Committee (IACUC) (AUP #2012-131). All surgery was performed under sodium pentobarbital anesthesia, and all efforts were made to minimize suffering. To produce tumors, RKO cells were harvested from subconfluent cultures by a brief exposure to 0.25% trypsin and 0.02% ethylenediaminetetraacetic acid. Trypsinization was stopped with medium containing 10% fetal bovine serum, and the cells were washed once in serum-free medium and resuspended in serum free medium. Only suspensions consisting of single cells with 90% viability were used for the injections. A xenograft was established by subcutaneous injection of the cells (3×10^6^) into the flanks of individual mice. Tumors were allowed to grow for 6 d until they were palpable. Mice were then randomized into two groups of 6 mice per group and dosed by oral gavage in corn oil 200 mg/kg/day (neutralized with an equimolar concentration of NaOH) of aspirin on every day for 14 d. The mice were weighed, and tumor size was measured every second day with calipers to permit calculation of tumor volumes: V = LW^2^/2, where L and W were length and width, respectively. After 14 d, the animals were sacrificed; final body and tumor weights were determined and plotted. At the end of the experiment, major visceral organs and tumors were collected and analyzed for Sp protein expression and induction of apoptosis using western blots and TUNEL staining respectively.

### Statistical analysis

Statistical significance of differences was determined by analysis of variance and student t-test, and the levels of probability were noted. All statistical tests were two-sided. IC_50_ values were calculated using non-linear regression analysis and expressed in µM, at 95% confidence intervals.

## Results

### Aspirin inhibits growth and induces apoptosis in colon cancer cells

**Figure 1:**
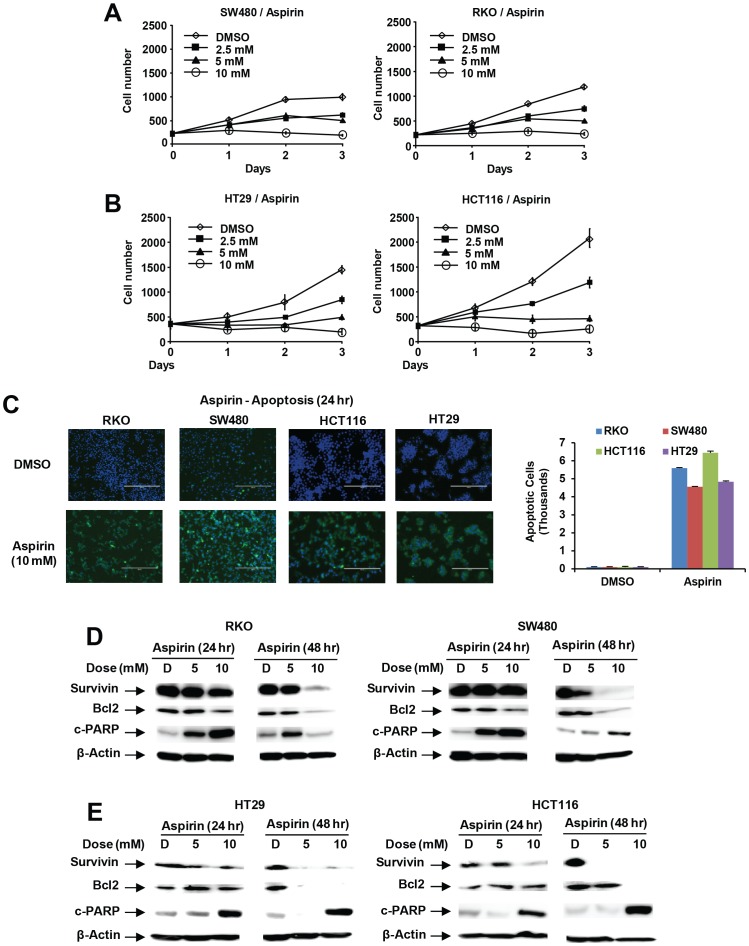
Aspirin inhibits colon cancer cell growth and induces apoptosis. Inhibition of SW480 and RKO (A) and HT29 and HCT116 (B) cell proliferation. Cells were treated with DMSO or 2.5–10 mM aspirin for 3 days, and cell numbers were determined as described in the Experimental Procedures. Induction of Annexin V staining (C) and apoptotic responses in RKO and SW480 (D) and HT29 and HCT116 (E) cells. Annexin V staining was determined as described in the Experimental Procedures. The expression of apoptotic proteins PARP cleavage was determined by western blot analysis of whole cell lysates as described in the Experimental Procedures. Results in (A) and (B) are means ± SE for 3 replicate determination for each treatment group, and significant (p<0.05) inhibition is indicated (*).

In this study, different concentrations (2.5–10 mM) of aspirin inhibited growth of SW480, RKO, HT29 and HCT116 cells over a period of 3 days (Figs. 1A and 1B), and IC_50_ values for aspirin-induced growth inhibition were in the range of 2.5–5 mM in all 4 cell lines. The high dose (10 mM) of aspirin was used to determine the proapoptotic effects of this compound after treatment for only 24 hr and the results show that aspirin increased Annexin V staining in all 4 colon cancer cell lines (Fig. 1C). The proapoptotic effects of aspirin (5 and 10 mM) were further investigated in colon cancer cells by determining changes in expression in the survival proteins bcl-2 and survivin and induction of caspase-dependent PARP cleavage. Aspirin induced a concentration- and time-dependent downregulation of survivin and bcl-2 and induced of PARP cleavage, a marker of apoptosis (Figs. 1D and 1E).

### Aspirin and salicylate downregulates Sp1, Sp3, Sp4 and Sp-regulated genes

**Figure 2:**
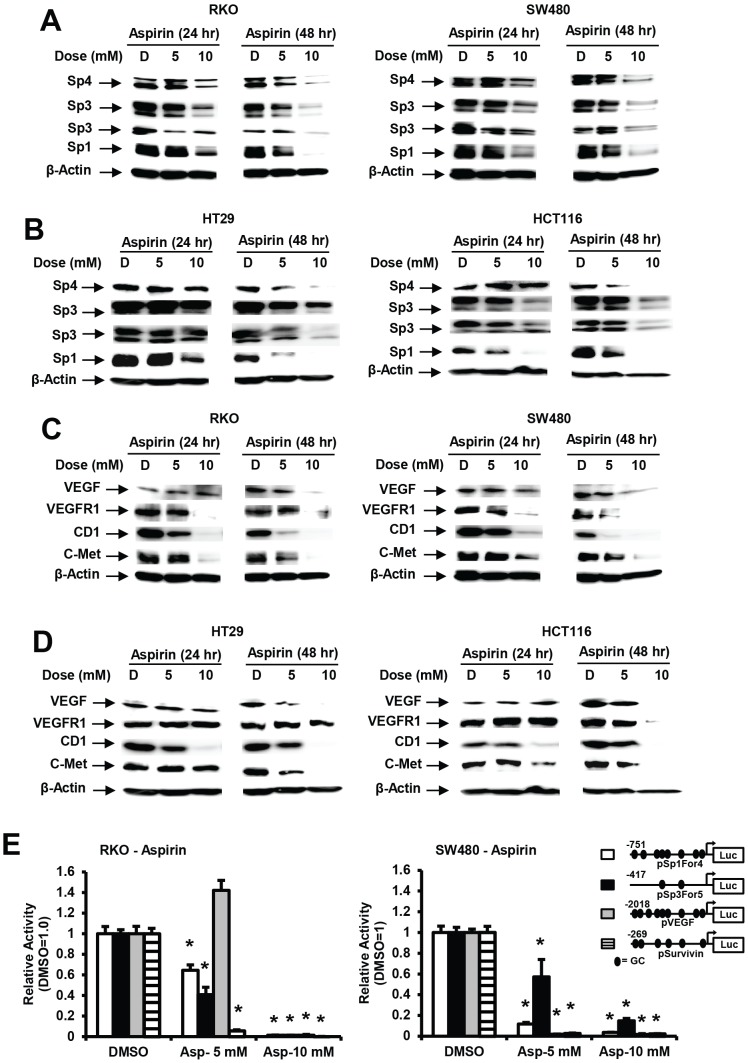
Aspirin decreases expressions of Sp1, Sp3, Sp4 and Sp-regulated gene products in colon cancer cells. Downregulation of Sp proteins in RKO and SW480 (A) and HT29 and HCT116 (B) and Sp-regulated gene products in RKO and SW480 (C) and HT29 and HCT116 (D) cells. Cells were treated with 5 or 10 mM aspirin for 24 or 48 hr, and whole cell lysates were analyzed by western blot analysis as described in the Experimental Procedures. Results are typical of duplicate experiments. (E) Aspirin decreases reporter gene activity. Cells were transfected with pSp1For4, pSp3For5, pVEGF and pSurvivin and treated with DMSO or aspirin (5 or 10 mM). Luciferase activity was determined as described in the Experimental Procedures. Results are expressed as means ± SE (3 replicates) and significant (p<0.05) inhibition is indicated (*).

Previous studies by RNA interference (RNAi) show that both survivin and bcl-2 are regulated by Sp1, Sp3 and Sp4 in cancer cells [17], [22], [24]–[26]. Therefore, RKO, SW480, HT29 and HCT116 cells were treated with 5 and 10 mM aspirin for 24 or 48 hr and western blot analysis showed that there was a concentration- and time-dependent decrease in Sp1, Sp3 and Sp4 proteins in all 4 cell lines (Figs. 2A and 2B). Moreover, treatment of RKO, SW480, HT-29 and HCT116 colon cancer cells with 5 or 10 mM aspirin for 24 or 48 hr also decreased expression of several gene products regulated by Sp1, Sp3 and Sp4 [24]–[29] and these include VEGF, VEGFR1, cyclin D1 and c-MET proteins (Figs. 2C and 2D), and the effects of aspirin on their expression were both concentration- and time-dependent.

We also investigated the effects of 5 and 10 mM aspirin on luciferase activity in RKO and SW480 cells transfected with constructs containing GC-rich promoter inserts from the Sp1 (pSp1For4, −751 to −20), Sp3 (pSp3For5, −417 to −38), VEGF (pVEGF, −2018 to +50), and survivin (pSurvivin, −269 to +49) genes (Fig. 2E). With the exception of the VEGF construct, 5 and 10 mM aspirin significantly decreased luciferase activity and these results correlated with the decreased expression of the corresponding Sp1, Sp3, VEGF and survivin proteins in RKO and SW480 cells. The increase in luciferase activity in RKO cells treated with 5 mM aspirin and transfected with pVEGF may be due to the relatively slow downregulation of this protein which was only observed after 48 hr at the 10 mM concentration, suggesting that other aspirin-induced pathways may also be activating VEGF.

**Figure 3:**
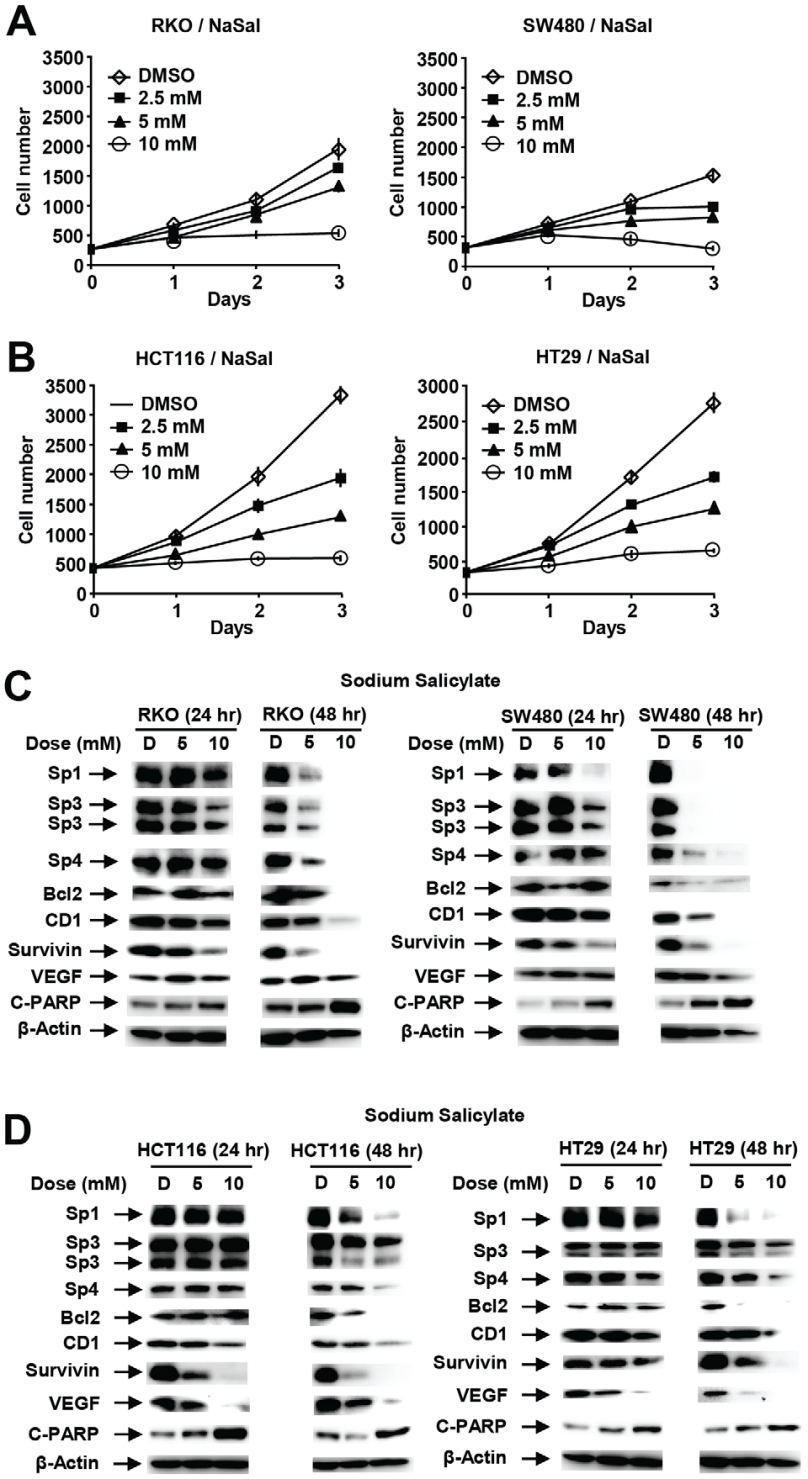
Salicylate inhibits colon cancer cell growth and downregulates Sp1, Sp3, Sp4 and Sp-regulated genes. Inhibition of RKO and SW480 (A) and HCT116 and HT29 (B) cell growth. Cells were treated with 2.5–10 mM sodium salicylate for up to 3 days, and cell numbers were determined as described in the Experimental Procedures. Protein downregulation in RKO and SW480 (C) and HCT116 and HT29 (D) cells. Cells were treated with 5 or 10 mM salicylate for 24 or 48 hr, and whole cell lysates were analyzed by western blots as described in the Experimental Procedures.

Aspirin is rapidly metabolized to salicylate [27] and the effects of salicylate on colon cancer cell growth, Sp proteins and Sp-regulated genes was also investigated (Fig. 3). Salicylate (sodium salt) (2.5–10 mM) also inhibited growth of all 4 cell lines (Figs. 3A and 3B), and the growth inhibitory effects were similar to that observed for aspirin (Figs. 2A and 2B). Growth inhibition was accompanied by the time-dependent downregulation of Sp1, Sp3, Sp4 and Sp-regulated gene products (bcl-2, cyclin D1, survivin and VEGF) in RKO and SW480 (Fig. 3C) and HCT116 and HT29 (Fig. 3D) cells. This pattern of responses for sodium salicylate was comparable to that observed for aspirin (Figs. 1 and 2) and similar effects were also observed for methyl salicylate (data not shown) which is also rapidly metabolized to salicylate.

### Aspirin-induced suppression of NFκB and β-catenin is due, in part, to Sp-downregulation

**Figure 4:**
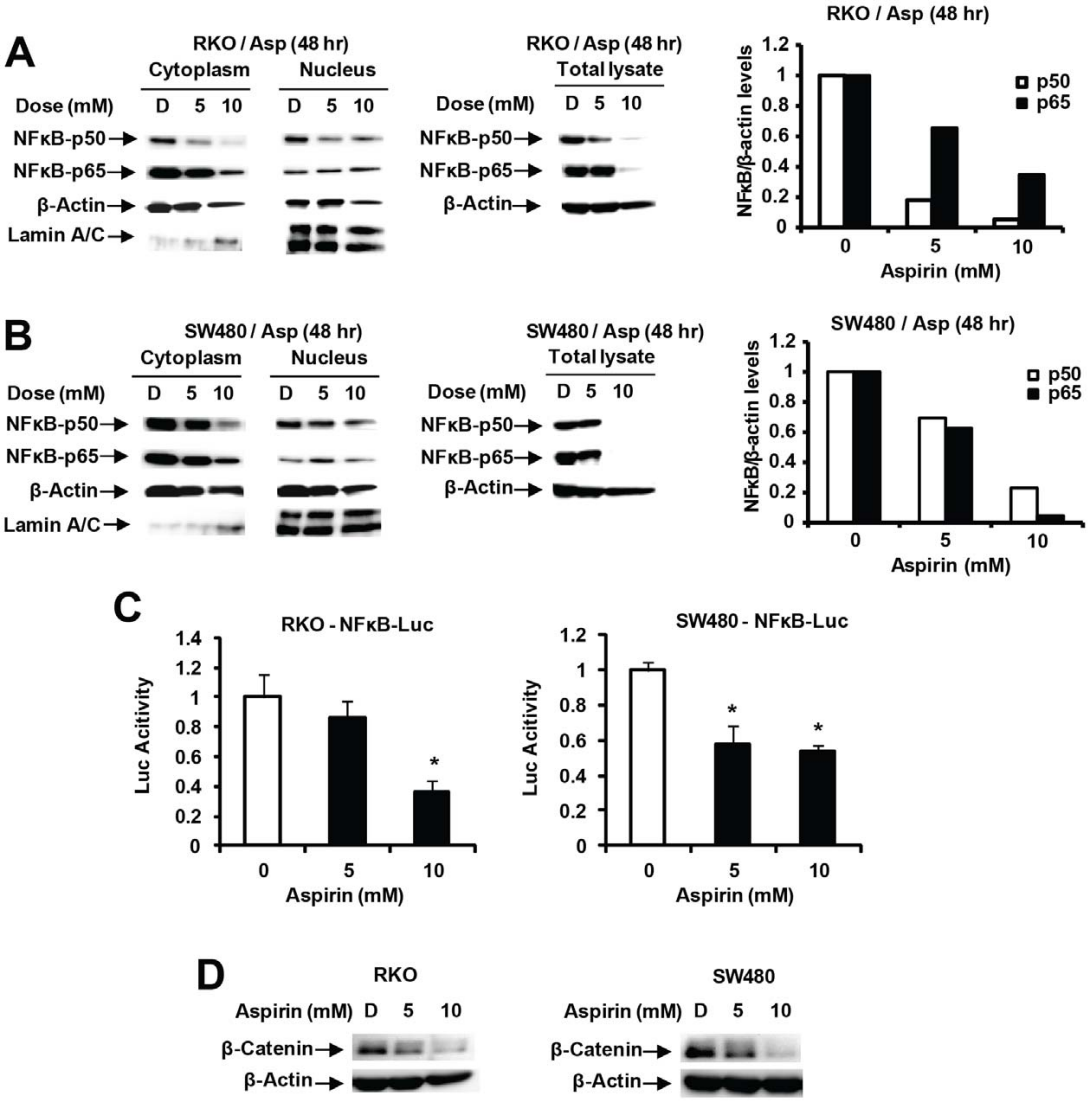
Aspirin decreases expression of NFκB and β-catenin in colon cancer cells. Decreased p65/p50 in RKO (A) and SW480 (B) cells. Cells were treated for 48 hr with 5 or 10 mM, and whole cell, nuclear and cytosolic extracts were analyzed by western blot analysis as described in the Experimental Procedures. Levels of p65 and p50 proteins (relative to β-actin) in whole cell lysates were quantitated from 3 replicate experiments and were significantly decreased by aspirin. (C) Aspirin decreases NFκB-luc. The construct was transfected into RKO and SW480 cells treated with DMSO or aspirin, and luciferase activity determined as described in the Experimental Procedures. Results are means ± SE (3 replicates) and significant (p<0.05) inhibition is indicated (*). (D) Downregulation of β-catenin. Cells were treated with 5 or 10 mM aspirin for 48 hr, and whole cell lysates were analyzed by western blots as described in the Experimental Procedures.

It has previously been reported that aspirin enhanced nuclear NFκB accumulation [13]–[15] and this was further investigated in RKO and SW480 cells treated with 5 and 10 mM aspirin for 48 hr. Levels of cytosolic p65 and p50 levels were decreased, whereas nuclear p65 and p50 levels were relatively unchanged after treatment with aspirin (Figs. 4A and 4B) and these results differed with previous studies [13]–[15]. Moreover, overall levels of nuclear proteins were <10% of cytosolic proteins and this was confirmed by western blot analysis of whole cell lysates from cells treated with 5 or 10 mM aspirin which showed that both p65 and p50 proteins were decreased in both cell lines within 48 hr after treatment with aspirin (Figs. 4A and 4B). Aspirin also decreased luciferase activity in SW480 and RKO cells transfected with a pNFκB-luc construct (Fig. 4C) and these results were consistent with the observed downregulation of both p65 and p50 proteins (Figs. 4A and 4B). Previous studies reported that aspirin decreased β-catenin expression in colon cancer cells [12] and our results confirmed that 5–10 mM aspirin also decreases β-catenin expression in RKO and SW480 colon cancer cells (Fig. 4D).

**Figure 5:**
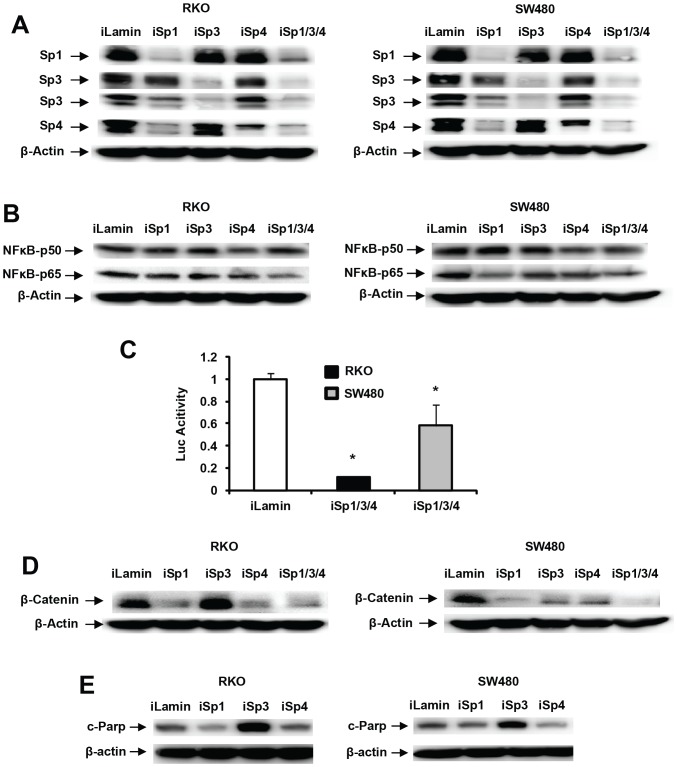
Knockdown of Sp1, Sp3 and Sp4 (alone and combined) by RNA interference. Knockdown of Sp1, Sp3, Sp4 and Sp1/3/4 (A) and p65/p50 (B) in colon cancer cells. Cells were transfected with various oligonucleotides, and whole cell lysates were analyzed by western blot analysis as outlined in the Experimental Procedures. (C) Knockdown of Sp1/3/4 (combined) inhibits NFκB-luc. Cells were transfected with iLamin (control) and iSp1/3/4 (combined oligonucleotides) and NFκB-luc, and luciferase activity was determined as described in the Experimental Procedures. Results are expressed as means ± SE (3 replicates), and significantly (p<0.05) decreased activity is indicated (*). Sp knockdown decreases β-catenin (D) and induces PARP cleavage (E). Cells were transfected with various oligonucleotides, and whole cell lysates were analyzed by western blots as described in the Experimental Procedures.

Since NFκB (p65) and the β-catenin promoters contain GC-rich Sp binding sites [28], [29], we used RNAi to determine the role of Sp1, Sp3 and Sp4 in regulation of p65, p50 and β-catenin in colon cancer cells. RKO and SW480 cells were transfected with small inhibitory RNAs targeting Sp1 (iSp1), Sp3 (iSp3), and Sp4 (iSp4) alone and in combination (iSp1/3/4). Figure 5A shows that each individual oligonucleotide decreased expression of its individual target (Sp1, Sp3 and Sp4) and iSp1/3/4 knocked down all three proteins. iSp1 also decreased expression of Sp4 (but not Sp3) and this is consistent with previous studies showing cross-regulatory interactions among the Sp transcription factors due to their GC-rich promoters [22], [25]. Figure 5B illustrates cell context-dependent differences in the effects of iSp1, iSp3 and iSp4 on p65 expression in RKO and SW480 cells where Sp1, Sp3 and Sp4 knockdown resulted in small changes in p65 in RKO cells and Sp1 and to a lesser extent Sp4 knockdown decreased p65 in SW480 cells. iSp1/3/4 (combined oligonucleotides) decreased p65 expression in both cell lines, whereas minimal effects were observed for p50. Thus, aspirin-induced downregulation of p50 (Figs. 4A and 4B) was Sp-independent. Figure 5C shows that iSp1/3/4 also decreased luciferase activity in RKO (90%) and SW480 (40%) cells transfected with the NFκB-luc construct, and the different effects of Sp knockdown in these cells suggest a more dominant role for Sp-dependent regulation of NFκB in RKO than SW480 cells. Sp knockdown (combined) also decreased β-catenin protein in RKO and SW480 cells and results of knockdown of individual Sp proteins suggest that Sp1 and Sp4 are the dominant transcription factors required for constitutive expression of β-catenin in RKO cells, whereas knockdown of Sp1, Sp3 and Sp4 decrease β-catenin protein levels in SW480 cells (Fig. 5D). Interestingly, the RNAi studies shows that PARP cleavage (apoptosis) is induced primarily after knockdown of Sp3 in both RKO and SW480 cells (Fig. 5E).

### Mechanism of aspirin-induced downregulation of Sp1, Sp3 and Sp4

**Figure 6:**
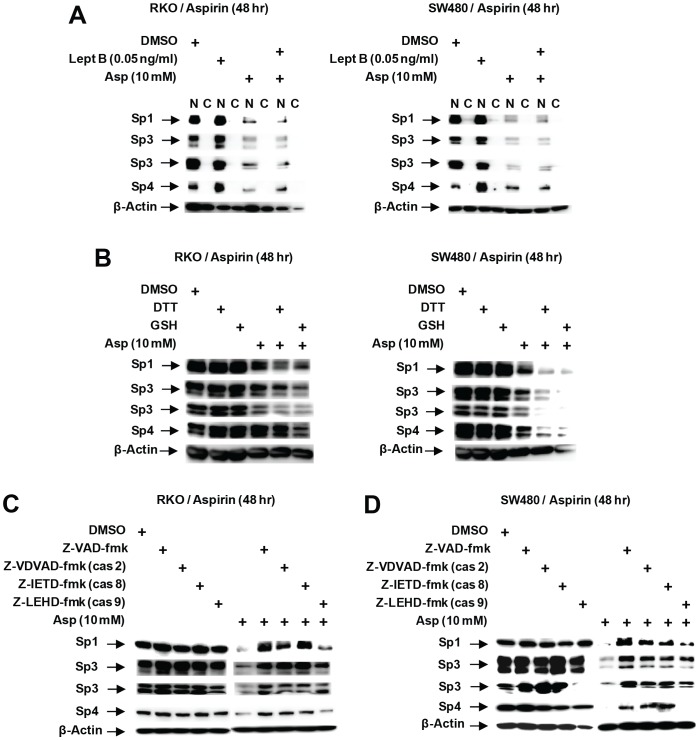
Mechanisms of aspirin-induced Sp protein degradation. (A) Effects of leptomycin B. Cells were treated with 10 mM aspirin in the presence or absence of leptomycin B for 48 hr, and whole cell lysates were analyzed by western blot analysis as described in the Experimental Procedures. Effects of antioxidants (B) and caspase inhibitors (C, D) on aspirin-induced Sp protein downregulation. Cells were treated with DMSO, aspirin alone or in combination with antioxidants or caspase inhibitors, and after 48 hr, whole cell lysates were analyzed by western blots as described in the Experimental Procedures.

Several pathways have been described for enhanced Sp degradation in cancer cell lines and these include activation of proteasomes, caspases and ROS [17]–[20], [22]–[26] and also a pathway which involves nuclear export of Sp1 followed by proteolytic degradation in the cytosol [30]. Western blot analysis of nuclear and cytosol fractions from RKO and SW480 cells shows that Sp1, Sp3 and Sp4 are localized in the nucleus and treatment with aspirin decreased Sp1, Sp3 and Sp4 protein levels in the nucleus without any apparent translocation to the cytosol (Fig. 6A). Similar results were observed after cotreatment with aspirin and the nuclear export inhibitor leptomycin B (combined), indicating that nuclear export of Sp1, Sp3 or Sp4 was not required for aspirin-induced downregulation of Sp1, Sp3 and Sp4. Other agents such as the nitro-NSAID GT-094 and betulinic acid decreased Sp proteins in RKO and SW480 cells through ROS-dependent induction of Sp transcriptional repressor ZBTB10 and this response was attenuated by antioxidants such as GSH or DTT [17], [31]; however, results in Figure 6B show that these antioxidants do not block aspirin-induced downregulation of Sp proteins. Results of preliminary studies show that aspirin-induced downregulation of Sp proteins was also not blocked by proteasome inhibitors (data not shown) but was affected by cotreatment with the pancaspase inhibitor Z-VAD-fmk. Figures 6C and 6D show that aspirin-induced Sp downregulation in RKO and SW480 cells was inhibited after cotreatment with the pancaspase inhibitor (Z-VAD-fmk), caspase-2 (Z-VDVAD-fmk), and caspase-8 (Z-IETD-fmk) inhibitors but only partially blocked with the caspase 9 (Z-LEHD-fmk) inhibitor.

**Figure 7:**
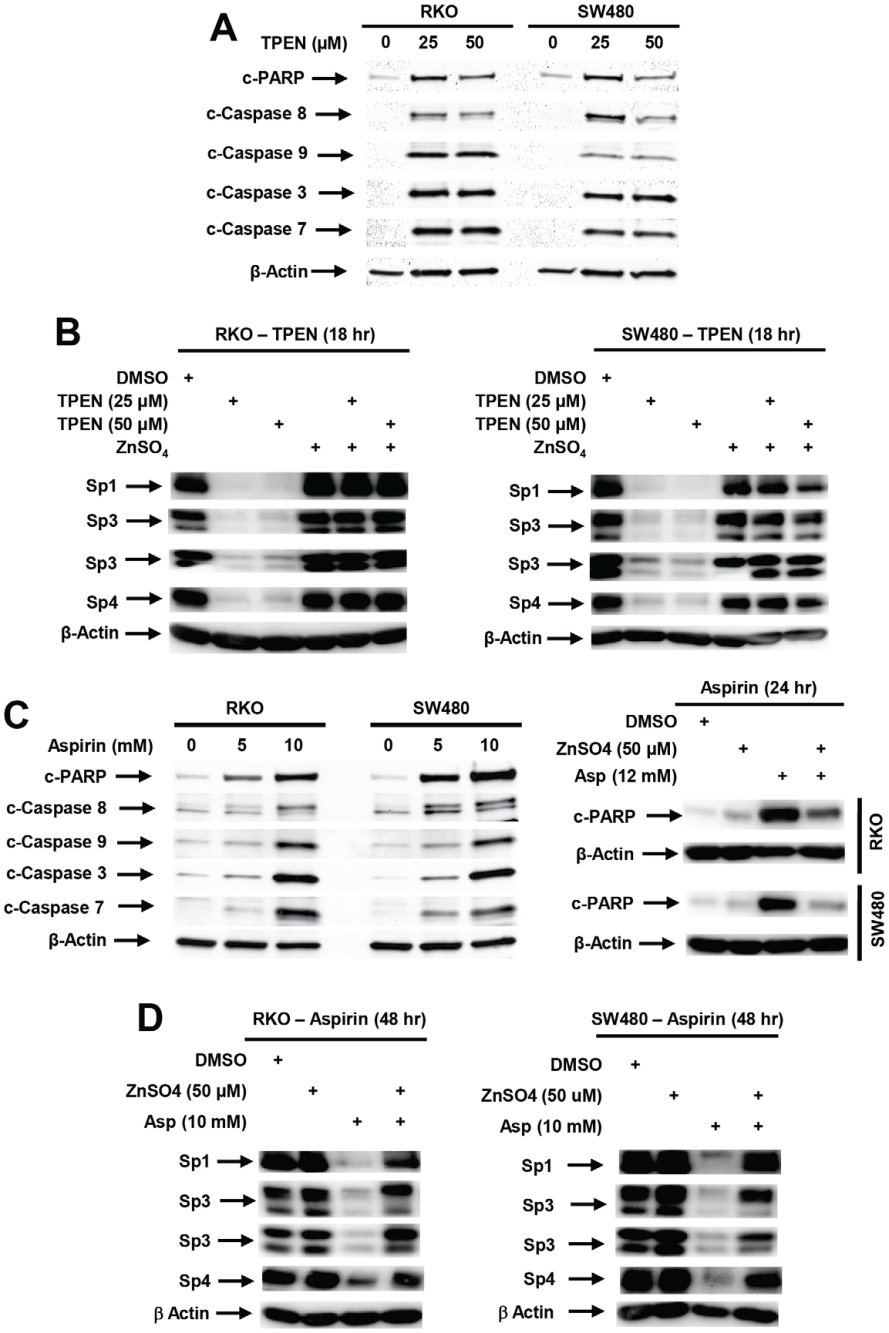
Zinc depletion results in induction of caspases and downregulation of Sp proteins. (A) TPEN induces activation (cleavage) of caspases. Cells were treated with 25 or 50 µM TPEN for 18 hr, and whole cell lysates were analyzed by western blots as described in the Experimental Procedures. (B) TPEN decreases Sp protein expression and zinc sulfate blocks the effects of TPEN on Sp downregulation. Cells were treated with 50 µM ZnSO_4_ for 18 hr, and whole cell lysates were analyzed by western blots. (C) Aspirin activates caspases. Cells were treated with aspirin for 48 hr, and whole cell lysates were analyzed by western blots as outlined in the Experimental Procedures. (D) Aspirin-induced PARP cleavage is blocked by ZnSO_4_. Cells were treated for 48 hr with aspirin alone or in combination with ZnSO_4_ and PARP cleavage was analyzed by western blots of whole cell lysates. (E) Effects of ZnSO_4_ on aspirin-induced downregulation of Sp proteins. RKO or SW480 cells were treated with 10 mM aspirin alone or in combination with 50 µM ZnSO_4_ for 48 hr, and whole cell lysates were analyzed by western blots as outlined in the Experimental Procedures.

Favier and coworkers [32], [33] previously reported in HeLa cells that zinc chelation by the permeable metal ion chelator TPEN activated multiple caspases (3-, 8- and 9-) and decreased expression of Sp1, Sp3 and Sp4 proteins that contain zinc ions in their catalytic sites. Treatment of RKO and SW480 cells with 25 or 50 µM TPEN for 18 hr induced PARP cleavage and activation (cleavage) of caspases 8, 9, 3, and 7 (Fig. 7A). The critical role of zinc depletion in mediating this response and downregulation of Sp proteins was confirmed by treating the colon cancer cells with TPEN alone or in combination with 50 µM ZnSO_4_ (Fig. 7B). TPEN decreased expression of Sp1, Sp3 and Sp4 and the addition of ZnSO_4_ completely reversed the TPEN-dependent downregulation of Sp1, Sp3 and Sp4 proteins. Like TPEN, aspirin also induced PARP cleavage and activation (cleavage) of caspases 8, 9, 3 and 7 (Fig. 7C). Aspirin-induced PARP cleavage was also inhibited by zinc sulfate (Fig. 7D); moreover, treatment of RKO and SW480 cells with aspirin alone or in combination with 50 µM ZnSO_4_ show that ZnSO_4_ also decreased aspirin-mediated downregulation of Sp1, Sp3 and Sp4 (Fig. 7E), thus confirming that aspirin, like TPEN, induces caspase-dependent cleavage of Sp proteins that is due to zinc ion depletion.

### *In vivo* studies

**Figure 8:**
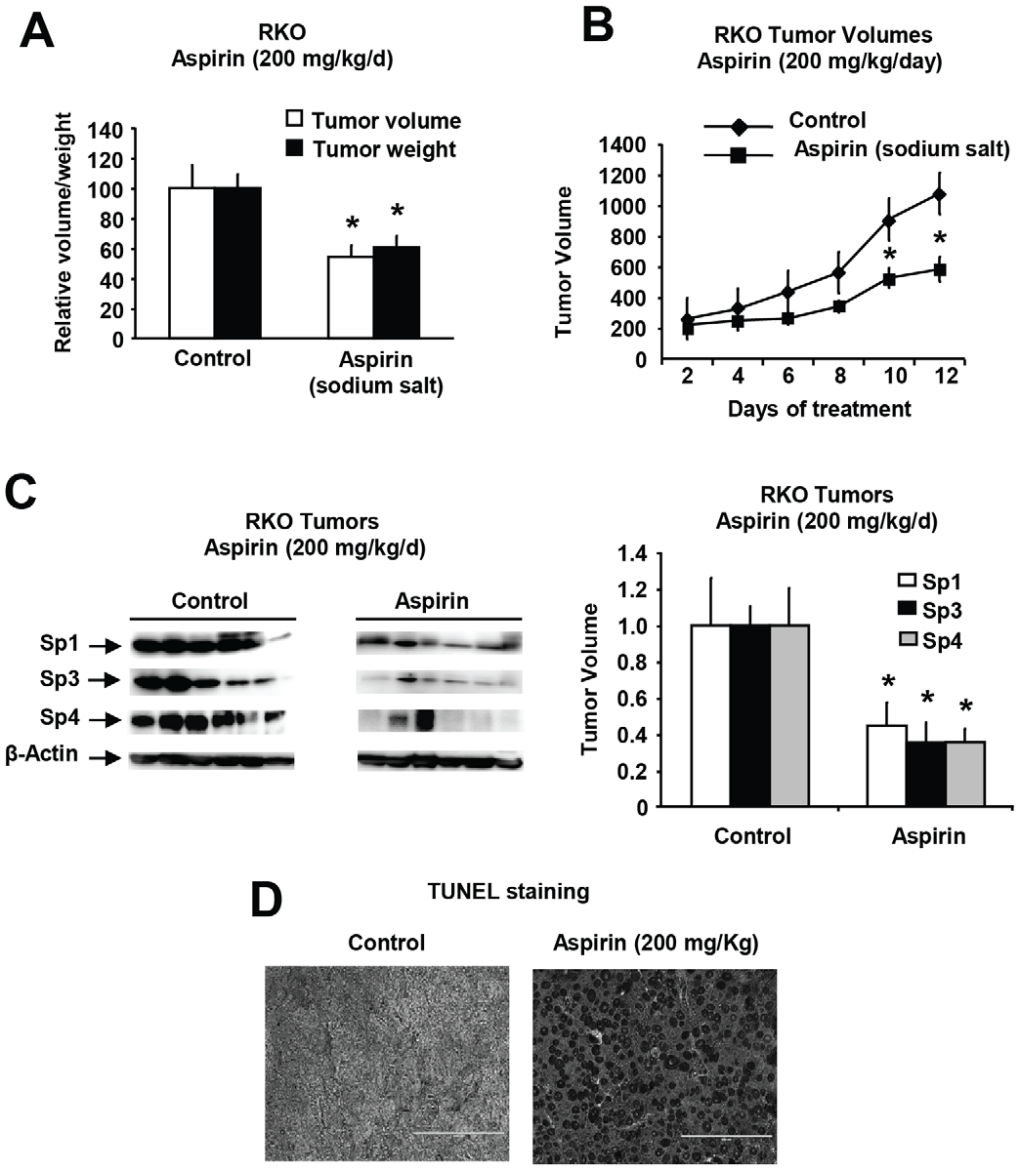
Aspirin inhibits colon tumor growth in athymic nude mice (xenografts). Inhibition of tumor weight (A) and volume (growth) (B) in athymic nude mice administered the sodium salt of aspirin. Athymic nude mice bearing RKO cells as xenografts were treated with the sodium salt of aspirin, and tumor volumes and weights were determined after sacrifice as described in the Experimental Procedures. (C) Expression of Sp1, Sp3 and Sp4 in colon tumors. Tumor lysates from solvent (control) and aspirin-treated mice were analyzed by western blot analysis as described in the Experimental Procedures. Expression of Sp1, Sp3 and Sp4 in aspirin-treated tumors compared to solvent (control)-treated tumors (set at 100%) was determined by densitometry, and β-actin was used to normalize protein expression. Results are means ± SE (6 replicates) and significant (p<0.05) inhibition of Sp1, Sp3 and Sp4 protein levels by aspirin is indicated (*). (D) Induction of apoptosis. Fixed tumor tissue from control and aspirin-treated mice were analyzed for TUNEL staining as outlined in the Experimental Procedures.

In athymic nude mice bearing RKO cells as xenografts, aspirin was administered when the tumors were initially palpable. The sodium salt of aspirin (200 mg/kg/d) was administered daily by oral gavage and this resulted in decreased tumor volumes and weights (Figs. 8A and 8B) and due to the lack of toxicity of the sodium salt, the treatment was continued for several days without any apparent signs of toxicity. Tumor lysates from control and treated animals were analyzed by western blot analysis for Sp1, Sp3 and Sp4, and levels were quantitated relative to β-actin and showed significant decreases in expression of Sp1, Sp3 and Sp4 in aspirin vs. control animals (Fig. 8C). Figure 8D shows increased apoptosis (TUNEL staining) in tumors from aspirin-treated mice compared to controls and these results complement *in vitro* studies showing that aspirin induced apoptosis in colon cancer cells (Fig. 1). These data confirm that aspirin-mediated inhibition of colon cancer cell and tumor growth was accompanied by downregulation of Sp transcription factors and this response contributes to the anticancer activity of aspirin.

## Discussion

Aspirin and other NSAIDs reduce the incidence and increase survival of colon cancer patients and similar results have been observed for aspirin as a chemotherapeutic agent for treatment of breast cancer patients [4]–[8]. These results indicate that aspirin has significant potential as a chemotherapeutic agent, although there is concern regarding the adverse gastrointestinal side-effects of aspirin [2], [3], [34] and in the future, these may be circumvented by using NO-NSAIDs. The effective doses of aspirin in most *in vitro* studies in colon cancer cells vary from 1–10 mM [9]–[15] and we observed significant inhibition of SW480, RKO, HT29 and HCT116 cell growth at ≤2.5 mM aspirin (Fig. 1) and 10 mM aspirin completely inhibited cell growth without causing seeded cells to detach. In children with autoimmune disease, treatment with aspirin (25 mg/kg) exhibited maximum serum concentrations of 5.2 mM aspirin with a range of 0.38–10.26 mM [27], suggesting that the dose range of aspirin used in this and many previous studies in colon cancer cells (1–10 mM) is within the range of serum levels achieved in studies using pharmacological doses of aspirin [27].

RNA interference studies in several cancer cell lines show that knockdown of Sp1, Sp3 and Sp4 (singly or in combination) results in decreased expression of growth promoting (EGFR, c-MET, cyclin D1), survival (bcl-2, survivin), angiogenic (VEGF and its receptors), and pro-inflammatory [p65 (NFκB)] genes or gene products [18], [21]–[26]. Decreased Sp-regulated genes is also accompanied by decreased cell growth and cell cycle progression and induction of apoptosis after knockdown of Sp proteins [20]–[26]. Moreover, several anticancer agents including NSAIDs and GT-094 (a NO-NSAID), curcumin, betulinic acid and synthetic triterpenoids, and arsenic trioxide decrease expression of Sp1, Sp3, Sp4 and Sp-regulated genes in cancer cells and these effects contribute to their anticancer activity [17], [18], [20], [35]. The reported growth inhibitory and antiangiogenic activity of aspirin and the downregulation of bcl-2 expression [9]–[11] suggested that one of the mechanisms of action of aspirin may also be due to downregulation of Sp1, Sp3 and Sp4 transcription factors. Results in Figures 1 and 2 show that aspirin induced a time- and concentration-dependent downregulation of Sp1, Sp3, Sp4 and Sp-regulated gene products in RKO, SW480, HT29 and HCT116 cells. There was some variability among the different cell lines in terms of their sensitivity to aspirin; however, after treatment for 48 hr, downregulation of Sp1, Sp3 and Sp4 was observed at concentrations of aspirin that were ≤5 mM (HT29 and HCT116 cells) or 5–10 mM (RKO and SW480 cells). These results, coupled with *in vivo* data showing that the sodium salt of aspirin decreased colon tumor growth in a xenograft model (RKO cells) in athymic nude mice and also decreased levels of Sp1, Sp3 and Sp4 proteins in tumors (Fig. 7), suggest that aspirin-induced downregulation of Sp proteins plays a role in the anticancer activity of this compound.

Aspirin-induced inhibition of β-catenin and NFκB have also been associated with the anticancer activity of aspirin [12]–[15], and we have previously identified that p65-NFκB expression was regulated by Sp transcription factors in colon and bladder cancer cells [22], [25]. One study reported that aspirin induced rapid phosphorylation and inactivation of β-catenin within 60 min after treatment and this was accompanied by decreased expression of several putative β-catenin-regulated gene products (e.g. cyclin D1 and c-MET) after treatment for 24–72 hr [12]. Our results confirm that aspirin also decreased expression of β-catenin protein (Fig. 4D); however, results of knockdown of Sp proteins by RNAi showed that β-catenin itself is an Sp-regulated gene, demonstrating that the effects of aspirin on β-catenin are also due to downregulation of Sp proteins.

It was also reported that aspirin increased nuclear p65 levels and induced apoptosis in colon cancer cells within 24 hr after treatment, and this former response has been linked to decreased expression of IκBα [13]–[15]. In contrast, our results show that aspirin treatment had minimal effects on nuclear p65 or p50 levels and the dominant effect was a dramatic decrease in p65 and p50 proteins in whole cell lysates (Figs. 4A and 4B). RNAi shows that p65 is an Sp-regulated gene (Fig. 5B) as previously reported in other cancer cell lines [22], [25]. The maximal (>50%) decrease was only observed in cells transfected with iSp1/3/4 (combined); however, the overall decrease in p65 in this experiment (Fig. 5B) was less than observed in other cancer cell lines [22], [25] and aspirin-induced inhibition of NFκB was only due, in part, to downregulation of Sp1, Sp3 and Sp4 transcription factors. Thus, although aspirin induces a dramatic downregulation of p65 and p50 in colon cancer cells, Sp protein downregulation contributes to decreased p65 levels, whereas effects on p50 are Sp-dependent. Results of transfections assays with an NFκB-luc contruct (Fig. 5C) also suggests that Sp1, Sp3 and Sp4 differentially affect NFκB-dependent transactivation in RKO and SW480 cells and this is currently being investigated. Aspirin-induced downregulation of p65 expression is maximal after treatment for 48 hr, whereas previous studies showed that IκBα expression was observed as early as 6 hr after treatment [13]. This suggests that aspirin-mediated inhibition of NFκB may be due to both short and long term effects on IκBα and p65, respectively, and this is currently being investigated.

The NO-NSAID GT-094 also decreased expression of Sp proteins and Sp-regulated genes in RKO and SW480 cells and this was due to induction of ROS and ROS-dependent downregulation of miR-27a and induction of the Sp transcriptional repressor ZBTB10 [17]. These responses were inhibited by antioxidants, whereas aspirin induced Sp downregulation was not affected by antioxidants (Fig. 6B); however, the pan-caspase inhibitor Z-VAD-fmk inhibited aspirin-induced effects on Sp1, Sp3 and Sp4 (Fig. 6D). Arsenic trioxide also induced caspase-dependent cleavage of Sp3 and Sp4 in bladder cancer cells [26] and retinoid (CD437)-induced Sp1 degradation was also caspase-dependent in EL-4 cells [36]. It has also been reported that zinc depletion induces apoptosis and decreases Sp1, Sp3 and Sp4 in cancer cell lines [32], [33], and we confirmed that aspirin-induced PARP cleavage and downregulation of Sp1, Sp3 and Sp4 was inhibited in RKO and SW480 cells cotreated with aspirin plus ZnSO_4_ (Figs. 7D and 7E). The physical interactions of zinc ions with aspirin have previously been observed [37], and functional interactions between zinc ions and aspirin in terms of zinc-induced neurotoxicity and the fetal toxicity have also been reported [38], [39]. Results of this study has identified a novel pathway for aspirin-induced effects on Sp1, Sp3 and Sp4 in colon cancer cells, and current studies are focused on specific enzymes and pathways associated with the effects of aspirin on zinc homeostasis.

Previous Studies on the pharmacokinetics of aspirin (25 mg/kg) administered to children with autoimmune disease showed that maximum serum concentrations of aspirin were 5.20 mM (range of 0.38–10.26 mM), whereas the maximum concentration of the major aspirin metabolite salicylate was 172 mM with a range of 59.8–312.2 mM [27]. Thus, pharmacologic doses of aspirin that give low mM serum concentrations can be accompanied by >30-fold higher concentrations of salicylate which are more than sufficient to inhibit colon cancer cell growth and decrease Sp1, Sp3, Sp4 and Sp-regulated genes (Fig. 3). Our studies with salicylate show that both aspirin and salicylate induced similar responses with comparable potencies in colon cancer cells (Figs. 1, 2, 3) and this is consistent with previous reports [3], [10], [11], [40]. The high salicylate/aspirin serum ratios observed in children administered pharmacological doses of aspirin (25 mg/kg) [27] suggests that the salicylate metabolite may be a major contributor to the reported chemotherapeutic effects of aspirin in colon cancer patients. Colon cancer patients on low dose aspirin therapy (75 mg/d; ca. 1 mg/kg/d) would exhibit maximum average serum concentrations of salicylate (6.8 mM) and aspirin (0.21 mM) sufficient to inhibit colon cancer cell growth and decrease expression of Sp1, Sp3, Sp4 and Sp-regulated genes (Fig. 3).

In summary, this study demonstrates that aspirin induces caspase-dependent proteolysis of Sp1, Sp3 and Sp4 proteins in colon cancer cells and tumors and, this was accompanied by downregulation of several Sp-regulated genes involved in cell proliferation, survival and angiogenesis. Aspirin-induced apoptosis and Sp downregulation was due to activation of nuclear caspases and perturbation of zinc homeostasis, and the mechanisms that regulate this pathway are unknown and are currently being investigated. Based on the similar effects of aspirin and salicylate as anticancer agents observed in Figures 1, 2, 3 and other studies [3], [10], [11], [40] and the high serum salicylate/aspirin ratios [27], the cancer chemotherapeutic effects of aspirin observed in human cancer studies [3]–[5], [7], [8] may primarily be due to the salicylate metabolite.
